# A highly specific and sensitive serological assay detects SARS-CoV-2 antibody levels in COVID-19 patients that correlate with neutralization

**DOI:** 10.1007/s15010-020-01503-7

**Published:** 2020-08-21

**Authors:** David Peterhoff, Vivian Glück, Matthias Vogel, Philipp Schuster, Anja Schütz, Philip Neubert, Veruschka Albert, Stefanie Frisch, Mara Kiessling, Philip Pervan, Maren Werner, Nicole Ritter, Leon Babl, Maria Deichner, Frank Hanses, Matthias Lubnow, Thomas Müller, Dirk Lunz, Florian Hitzenbichler, Franz Audebert, Viola Hähnel, Robert Offner, Martina Müller, Stephan Schmid, Ralph Burkhardt, Thomas Glück, Michael Koller, Hans Helmut Niller, Bernhard Graf, Bernd Salzberger, Jürgen J. Wenzel, Jonathan Jantsch, André Gessner, Barbara Schmidt, Ralf Wagner

**Affiliations:** 1 Institute for Medical Microbiology and Hygiene, University of Regensburg, Regensburg, Germany; 2 Institute for Clinical Microbiology and Hygiene, University Hospital Regensburg, Regensburg, Germany; 3 Department for Infection Control and Infectious Diseases, University Hospital Regensburg, Regensburg, Germany; 4 Emergency Department, University Hospital Regensburg, Regensburg, Germany; 5 Department of Internal Medicine II, University Hospital Regensburg, Regensburg, Germany; 6 Department of Anesthesiology, University Hospital Regensburg, Regensburg, Germany; 7 Praxiszentrum Alte Mälzerei, Regensburg, Germany; 8 Institute of Clinical Chemistry and Laboratory Medicine, Transfusion Medicine, University Hospital Regensburg, Regensburg, Germany; 9 Department of Internal Medicine I, University Hospital Regensburg, Regensburg, Germany; 10 Institute of Clinical Chemistry and Laboratory Medicine, University Hospital Regensburg, Regensburg, Germany; 11 Kreisklinik Trostberg, Trostberg, Germany; 12 Center for Clinical Studies, University Hospital Regensburg, Regensburg, Germany

**Keywords:** SARS-CoV-2, COVID-19, Antibody test, ELISA, Serology, Virus neutralization, Assay validation, Spike protein, S protein, Receptor binding domain

## Abstract

**Objective:**

The severe acute respiratory syndrome coronavirus 2 (SARS-CoV-2) pandemic challenges national health systems and the global economy. Monitoring of infection rates and seroprevalence can guide public health measures to combat the pandemic. This depends on reliable tests on active and former infections. Here, we set out to develop and validate a specific and sensitive enzyme linked immunosorbent assay (ELISA) for detection of anti-SARS-CoV-2 antibody levels.

**Methods:**

In our ELISA, we used SARS-CoV-2 receptor-binding domain (RBD) and a stabilized version of the spike (S) ectodomain as antigens. We assessed sera from patients infected with seasonal coronaviruses, SARS-CoV-2 and controls. We determined and monitored IgM-, IgA- and IgG-antibody responses towards these antigens. In addition, for a panel of 22 sera, virus neutralization and ELISA parameters were measured and correlated.

**Results:**

The RBD-based ELISA detected SARS-CoV-2-directed antibodies, did not cross-react with seasonal coronavirus antibodies and correlated with virus neutralization (*R*^2^ = 0.89). Seroconversion started at 5 days after symptom onset and led to robust antibody levels at 10 days after symptom onset. We demonstrate high specificity (99.3%; *N* = 1000) and sensitivity (92% for IgA, 96% for IgG and 98% for IgM; > 10 days after PCR-proven infection; *N* = 53) in serum.

**Conclusions:**

With the described RBD-based ELISA protocol, we provide a reliable test for seroepidemiological surveys. Due to high specificity and strong correlation with virus neutralization, the RBD ELISA holds great potential to become a preferred tool to assess thresholds of protective immunity after infection and vaccination.

**Electronic supplementary material:**

The online version of this article (10.1007/s15010-020-01503-7) contains supplementary material, which is available to authorized users.

## Introduction

Coronaviruses (CoV) are known to cause respiratory diseases in humans. The alphacoronaviruses HCoV-229E and HCoV-NL63 as well as the betacoronaviruses HCoV-OC43 and HCoV-HKU1 circulate seasonally in humans and cause common colds [1]. In contrast, the severe acute respiratory syndrome-related coronavirus 1 (SARS-CoV-1), the Middle East respiratory syndrome-related coronavirus (MERS-CoV) and SARS-CoV-2 are zoonotic betacoronaviruses and can cause life-threatening severe respiratory distress syndromes and pandemics [2].

The immune response to the seasonal CoVs and SARS-CoV and MERS-CoV has been studied intensively [3, 4]. They are able to trigger a humoral immune response that correlates with disease severity [3]. Of note, mild infections resulted in short-lived and very low antibody titers near the detection limit. This loss of humoral immunity has been linked to the occurrence of (re-)infections with seasonal CoVs [3].

The current SARS-CoV-2-triggered COVID-19 pandemic started in December 2019 in Wuhan, Hubei province of China and has led with August 2020 to > 18 million confirmed infections [5]. Diagnosis of COVID-19 relies on PCR testing of respiratory specimen [6]. However, to assess whether a patient had recovered from a previous infection serological analyses are required. Serological surveys for SARS-CoV-2 antibody responses are not only important to monitor how much of a given population has been infected, but also serology will be required to assess vaccine responses. Finally, serology will be key to identify the quality of convalescent plasma that can be applied within clinical (compassionate) trials [7].

To allow for reliable serology, specific and sensitive assays are urgently needed. Here, we provide and validate a robust and simple ELISA protocol which is based on the SARS-CoV-2 receptor-binding domain (RBD). With this protocol, we performed a cross-sectional analysis of sera from PCR-proven COVID-19 patients. We demonstrate correlation of the ELISA test results with neutralizing antibody levels in a neutralization assay using a clinical isolate.

## Materials and methods

### Patient samples

The SARS-CoV-2 ELISA was validated using pseudonymized samples from patients aged older than 18 years from the diagnostic repository of the Institute of Clinical Microbiology and Hygiene, University Hospital Regensburg, originating from summers of 2016–2019. Potential cross-reactivity of the ELISA protocol was analyzed using sera from patients with PCR-proven seasonal coronavirus infections and detectable antibody reactivity against seasonal coronavirus antigens. Sensitivity of the protocol was quantified with sera from patients with PCR-proven SARS-CoV-2 infection at different time points. This procedure was approved by the ethical commission of the Faculty for Medicine, University of Regensburg, Regensburg, Germany (ref. no. 20-1854-101).

### Design of recombinant proteins

S-protein sequences used for sequence analysis and gene synthesis are given in Table S1. Phylogenetic analysis, synthesis and cloning of the S protein variants used in this study (Fig. S1) are described in the supplementary materials and methods.

### Protein structure analysis and visualization

Protein structures were visualized and analyzed by Pymol (LLC Schrodinger) using the structural data from the Protein Data Bank (https://www.rcsb.org) repository using entry codes 6vsb and 6m17.

### Protein production and purification

Proteins were expressed in Expi293 cells (Thermo Fisher Scientific) in different scales using the commercial ExpiFectamine™ system. Affinity purification of the proteins and quality controls are described in the supplementary materials and methods.

### Line blot assay

CoV line blot assay (beta-version of *recom*Line Coronavirus IgG assay) was performed as described in the manufacturer’s protocol. Line blots were evaluated by visual inspection and using *recom*Scan software (Mikrogen Diagnostik, Neuried, Germany).

### Enzyme-linked immunosorbent assay

A detailed ELISA protocol is provided in the supplementary materials and methods. Commercial Anti-SARS-CoV-2-ELISA IgG (EUROIMMUN, Lübeck, Germany) was performed according to the manufacturer’s recommendations.

### Virus isolation and virus load quantification

SARS-CoV-2 isolation from respiratory specimen, determination of the viral loads using quantitative SARS-CoV-2 real-time RT-PCR (RT-qPCR) and 50% tissue culture infective dose (TCID_50_) are described in the supplementary materials and methods.

### Virus neutralization assay

Virus neutralization assay using SARS-CoV-2 from respiratory specimen is described in the supplementary materials and methods.

### Data evaluation and curve fitting

Experimental data were evaluated and plotted using GraphPad Prism (GraphPad Prism version 8.4.2 for Windows, GraphPad Software, San Diego, California USA).

## Results

**Fig. 1:**
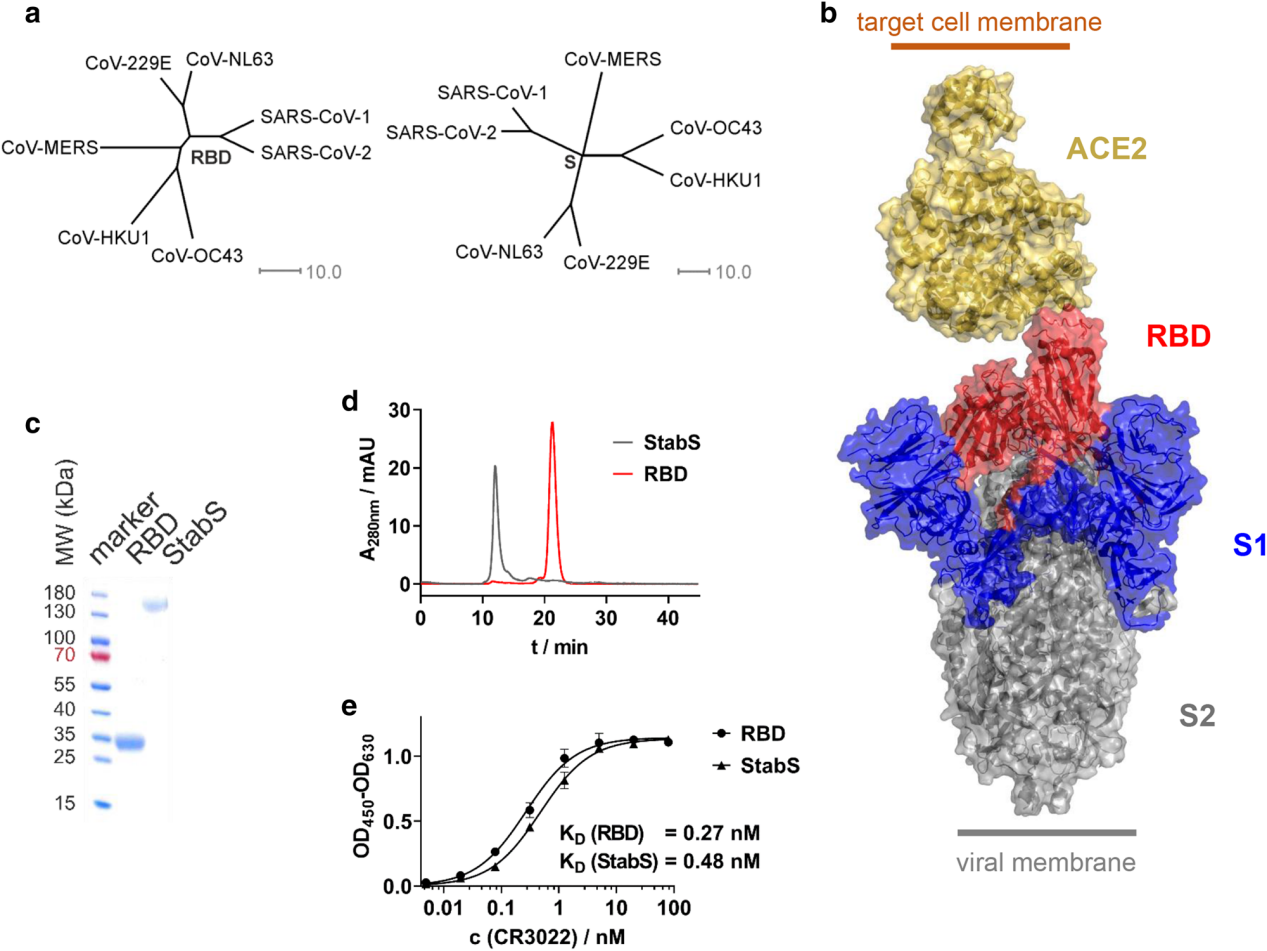
Antigens used in SARS-CoV-2 ELISA. **a** Phylogenetic trees calculated for the RBD and complete S-protein of clinically relevant members of the coronavirus family. Trees are calculated on the basis of percent identity between each pair of sequences in the respective alignment. **b** Structure of the S-protein ectodomain in complex with its receptor, angiotensin-converting enzyme 2 (ACE2). **c** Reducing SDS-PAGE (linear gradient of 8–16% polyacrylamide) of the purified RBD and StabS protein. **d** Size exclusion chromatography of the purified RBD and StabS protein. **e** ELISA titrations of SARS-CoV-1 and SARS-CoV-2-binding antibody CR3022 against immobilized StabS and RBD [OD_450–630_: optical density (OD) at 450 nm after background subtraction at 630 nm]. Resulting dissociation constants (*K*_D_) are given in the diagram

S-protein’s RBD has been shown to be SARS-CoV-1’s Achilles’ heel [8]. Due to the homology between the S-proteins as well as the RBDs of SARS-CoV-1 and SARS-CoV-2 (Fig. 1a), this may also hold true for the virus driving the current pandemic. This is why we decided to establish an ELISA protocol, which uses the SARS-CoV-2 RBD and stabilized ectodomain (StabS [9]) as antigens (Fig. 1b). To promote robust production of the two recombinant proteins, coding sequences were codon-optimized and the autologous S-protein signal peptide was replaced by a minimal version of the tissue plasminogen activator (tPA) signal peptide developed in our lab. RBD yielded 70 mg/l supernatant for the RBD and 14 mg/l supernatant for the StabS protein. Purity and homogeneity were verified by reducing SDS-PAGE and size exclusion chromatography (Fig. 1c, d).

A number of SARS-CoV-2 serology ELISA protocols have been published recently [10–15]. In our protocol, the antigens were directly absorbed to the plate’s plastic surface. To control protein integrity, we used our ELISA protocol in combination with the structure-dependent monoclonal anti-RBD antibody CR3022 [16–18]. High affinity binding as reflected by a *K*_D_ of 0.27 nM for the RBD protein and 0.48 nM for StabS as well as a strong absorption (OD_450–630_ > 1 at saturation after 4 min of development time) in both cases demonstrated sufficient amounts of well-folded protein (Fig. 1e). We used CR3022 at saturation concentration to test the stability of RBD-coated plates, which had been coated up to 8 days earlier. No performance loss was detected during this period when plates were stored at room temperature in PBS-T (Fig. S2).

We used our ELISA to screen for anti-S and anti-RBD antibodies in sera from COVID-19 patients and controls. In a first step, we quantified anti-RBD responses and anti-S responses in 22 sera, which displayed differential ELISA signals (Fig. S3).

We found that antibody responses against StabS correlated very well with antibody response to RBD as determined by correlation of OD_450–630_ at 1:100 dilution, area under the curve (AUC), effective concentration at 50% signal (EC_50_) and titer (Fig. S4). Moreover, comparable signal strengths in these analyses suggest that RBD is a key immunogenic determinant of anti-S responses (Fig. S3). Therefore, anti-RBD antibody levels represent a valuable surrogate for testing anti-S-directed antibody responses.

High-throughput titration of sera for the purpose of determining end point titers or calculating EC_50_ values—in particular in seroepidemiological surveillance studies on broader population scale—is a time- and material-consuming procedure. Thus, we tested whether OD_450–630_ at 1:100 dilution of sera correlate with standard serum characteristics such as area under the curve (AUC), effective concentration at 50% signal (EC_50_) and titer. This analysis revealed that 1:100 dilution of sera reflects total serum antibody responses (Fig. S5). Therefore, we used single-point measurements for all further assays.

**Fig. 2:**
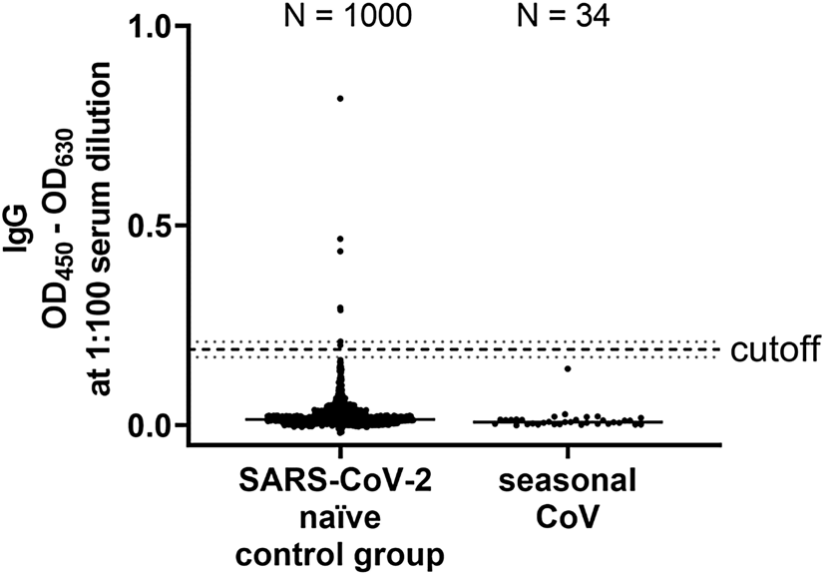
Assay specificity of RBD ELISA. To define the specificity of the assay, 1000 sera isolated in the summers of 2016 and 2018 (SARS-CoV-2 naïve control group) and 34 sera of patients with seasonal corona virus infection (seasonal CoV) were measured (median is shown). The cutoff is set at six standard deviations above the mean of the reference panel (borderline ± 10% cutoff)

Next, we wanted to establish cutoff values for anti-RBD-directed IgG, IgA and IgM antibody responses following the requirements for validation of diagnostic assays in clinical virology [19]. For that purpose, we measured 190 SARS-CoV-2 naïve sera that had been collected before the current SARS-CoV-2 pandemic (Fig. S6). As recently proposed by Okba et al*.* [11], we used the mean of the background signals plus six standard deviations (SD) to define the cutoff value. Using these parameters, we determined the specificity of our assay by measuring 1000 independent SARS-CoV-2 infection naïve sera. We obtained a false positive rate of 7 out of 1000 sera, corresponding to a specificity of 99.3% (Fig. 2).

To further verify the specificity of our ELISA, we tested whether sera from patients, that suffered from infection with seasonal CoVs, cross-react with our assay. For that purpose, we retrieved in a total of 43 sera from patients that had PCR-proven seasonal corona virus infection from our diagnostic repository. In a commercial line blot assay, 34 sera out of 43 scored positive for seasonal CoV-specific antibodies (Table S2). We used these 34 sera to further validate our RBD ELISA. Of utmost importance, none of these seasonal coronavirus sera showed any cross-reactivity in our ELISA (Fig. 2).

To verify precision and reproducibility of our ELISA [19], we used a minimal panel of seasonal (*N* = 5), naïve (*N* = 15), weakly IgG reactive (*N* = 10) and strongly IgG reactive (*N* = 10) sera. For weakly IgG reactive sera, the relative standard deviation (*σ*_rel_) was 3.98% for IgG, 1.72% for IgM and 7.15% for IgA; for strongly reactive IgG positive sera *σ*_rel_ was determined to be 0.16% for IgG, 0.22% for IgM, and 2.73% for IgA. Combined inter-assay and inter-operator variabilities for weakly IgG reactive sera was determined as a *σ*_rel_ of 10.26% and for strong IgG reactive sera *σ*_rel_ was calculated to be 4.12%.

**Fig. 3:**
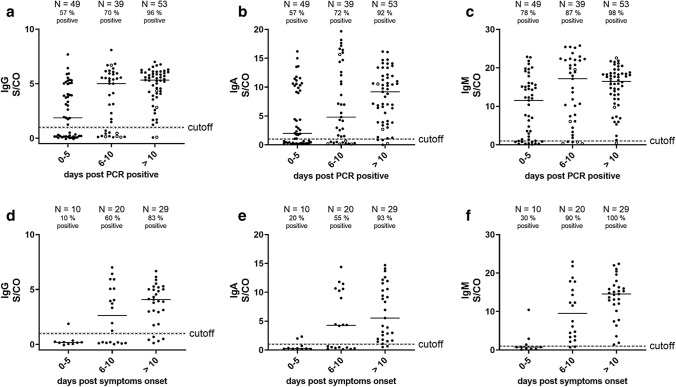
Serological testing of COVID-19 patients using RBD ELISA. **a**–**c** Serum reactivities of different immunoglobulin isotypes at 1:100 dilution of 144 sera of different time points after positive SARS-CoV-2 RT-qPCR, 1 per patient and time point (S/CO = signal/cutoff). Sampling of sera > 10 days post-PCR positive ranges from 11 to 29 days. Sera from patients with < 300 RNA copies/ml in respiratory specimen are shown as open circles, all others as closed circles. **d**–**f** Serum reactivities at 1:100 dilution (S/CO values) of 59 sera of different time points after onset of symptoms (subset of values from **a**–**c** with known case history), one per patient and time point

To determine the sensitivity of our assay, we analyzed sera from patients suffering from COVID-19 (*N* = 144), assuming that after symptomatic SARS-CoV-2 infection eventually all subjects develop antibodies. We quantified the anti-RBD IgG responses, which correlated well (*R*^2^ = 0.8812, Spearmen’s *ρ* = 0.917, *p* value < 0.0001) with anti-SARS-CoV-2 responses measured using a commercial IgG ELISA (EUROIMMUN, Fig. S7) that has been validated recently [20]. Next, we determined the IgM, IgG and IgA levels at different time frames after the first detection of SARS-CoV-2 RNA by RT-qPCR in our cohort (proven infection; Fig. 3a–c). At > 10 days after proven infection, these assays displayed sensitivities of 92% for IgA, 96% for IgG and 98% for IgM. However, we found that the antibody responses were already remarkably elevated at early time points after proven infection and, in concordance with previous findings, did not display a steady increase over time [21]. We speculated that infection preceded virus testing by several days. For 41% of the subjects (*N* = 59), the time point of symptom onset was available and we could calculate the average period between symptom onset and detection of SARS-CoV-2 RNA by RT-qPCR (5.7 days; Fig. S8). This suggests that the majority of patients who were subjected to PCR testing had already suffered from COVID-19 for 5.7 days. Therefore, an analysis of anti-RBD responses in relation to days after symptom onset should result in lower anti-RBD responses at early time points and a steady increase of antibody levels over time. Indeed, in the subgroup of patients where the time point of symptom onset was available, we detected very low antibody responses early after symptom onset and a steady increase in all anti-RBD antibody isotypes (Fig. 3d–f).

**Fig. 4:**
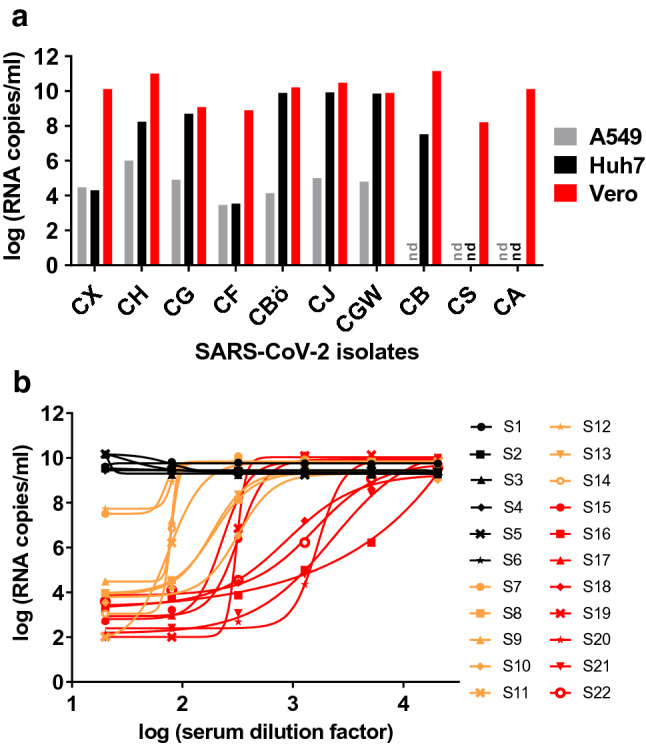
Virus isolation and neutralization assay. **a** Isolation of ten SARS-CoV-2 strains from respiratory specimen using three different cell lines (A549, derived from lung carcinoma; Huh7, hepatocyte-derived carcinoma; Vero, kidney epithelial cells from African green monkey; nd = not done). Viral loads in cell culture supernatants were determined using RT-qPCR. **b** After titration of the cytopathic SARS-CoV-2 strain CA, Vero cells were infected at a multiplicity of infection (MOI) of 0.05. Prior to infection, the virus was incubated with dilutions of 22 serum samples (fourfold serial dilutions starting at 1:20). Of these, six (S1–S6, black), eight (S7–S14, orange), and eight (S15–S22, red) samples displayed negative, medium, and high SARS-CoV-2 IgG reactivities, respectively, in the RBD-based ELISA. Two days post-infection, viral loads were determined in cell culture supernatants using RT-qPCR

Neutralizing antibodies correlate with protection against several pathogens. Since we used the RBD of SARS-CoV-2, we expected the serum reactivity measured in our ELISA to correlate with virus neutralization due to sterical hindrance of RBD’s binding to its receptor ACE2 (Fig. 1b). In a first step, we isolated ten SARS-CoV-2 strains from respiratory specimen of COVID-19 patients. Of three different cell lines, kidney epithelial cells (Vero) supported virus propagation more efficiently than hepatocarcinoma-derived Huh-7 cells and lung carcinoma-derived A549 cells (Fig. 4a). For this reason, Vero cells were selected for neutralization experiments, using a highly replicative and cytopathic SARS-CoV-2 isolate (strain CA).

**Fig. 5:**
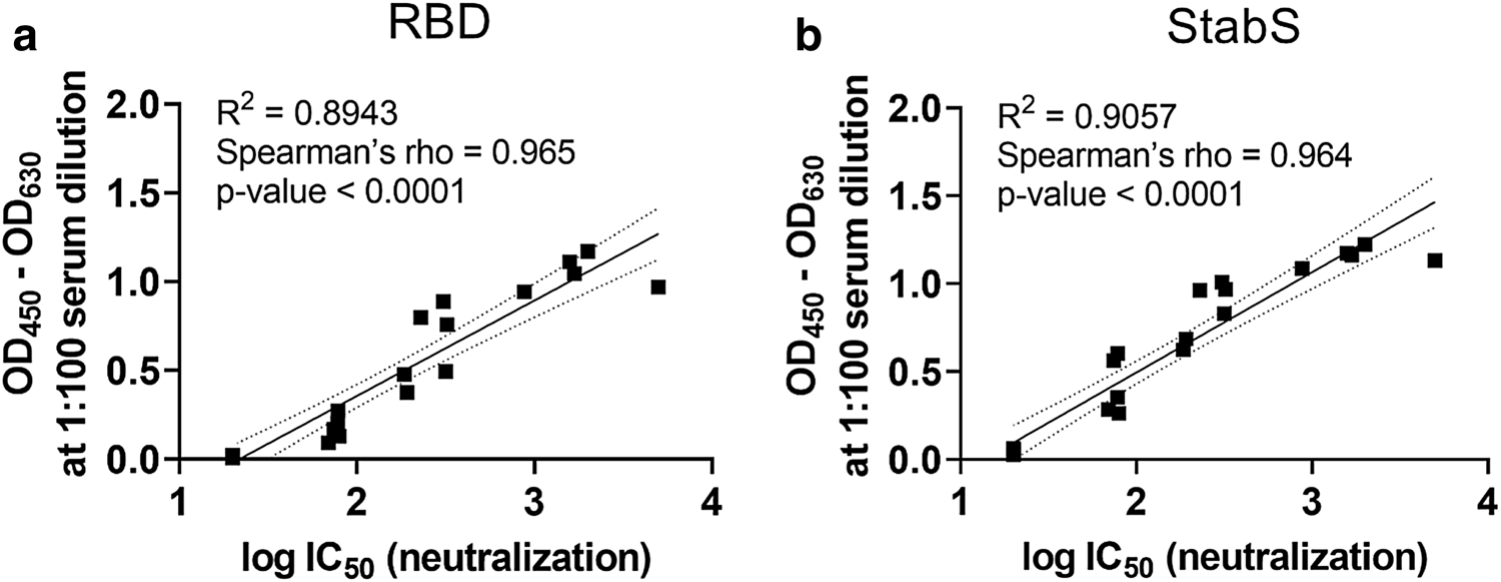
Correlation of antibody levels with SARS-CoV-2 neutralization. Correlation of OD from IgG ELISA measurements at 1:100 serum dilution with log IC_50_ values obtained from the neutralization experiments for a panel of 22 reference sera using RBD (**a**) and StabS (**b**) as an antigen. Coefficients of determination (*R*^2^), Spearmen’s *ρ*, *p* value, linear regression line (solid line) and 95% confidence intervals (dashed line) are given in the diagrams

Prior to infection of Vero cells, the virus was incubated with serial dilutions of the 22 sera, which displayed differential ELISA titrations (Fig. S3). After removal of the inoculum at 12–24 h post-infection, viral loads were determined in cell culture supernatants at 48 h post-infection using SARS-CoV-2 RT-qPCR. All sera which were negative in the ELISA (*N* = 6) did not inhibit virus entry and replication (Fig. 4b). In contrast, sera with borderline or positive ELISA values reduced SARS-CoV-2 viral loads by two (*N* = 2) to six log_10_ (*N* = 14). Notably, the reduction of viral loads showed a varying pattern, from a sudden to a more gradual inhibition of viral replication. Neutralization capacity was more pronounced in sera with high ELISA values. Most importantly, neutralization titers (given as IC50 values) correlated strongly with anti-RBD (*R*^2^ = 0.8943, Spearmen’s *ρ* = 0.965, *p* value < 0.0001) and anti-StabS (*R*^2^ = 0.9057, Spearmen’s *ρ* = 0.964, *p* value < 0.0001) antibody levels (Fig. 5a, b and Fig. S9a–f).

## Discussion

We established and validated a protocol for an easy-to-perform, robust and sensitive ELISA for detection of S-directed SARS-CoV-2 antibody responses in sera that correlate with SARS-CoV-2 virus neutralization.

As reported for a commercial assay our ELISA is highly specific (99.3%) as well, and sensitivity is dependent on the time point after infection when the serum sample is taken [20]. In concordance with findings of others, we detected robust antibody levels at approximately 2 weeks after onset of symptoms [22, 23]. Moreover, our data suggest that seroconversion for all Ig isotypes requires at least 6–10 days after onset of symptoms. In our cohort, IgM levels tend to appear first, followed by IgG conforming to earlier reports on SARS-CoV-2 Ig responses [21, 24–28]. Of note, we detected a late rise of the median IgA levels in our cohort. It is possible that this is an etiopathologic predictor of COVID-19 [29]. Taken into account, that the cohort of our cross-sectional study contained only symptomatic COVID-19 cases, seroconversion rates in oligo- or asymptomatic patients, the persistence of antibody levels and the role of antibodies in mucosal fluids warrant further investigation. Longitudinal epidemiological surveillance studies to address these questions are urgently needed. We provide an assay that can be easily implemented to quantify antibody responses in COVID-19.

The presence of RBD-directed antibodies, as determined by the herein described approach, correlate with SARS-CoV-2 neutralization. These findings strongly suggest that anti-RBD antibodies may confer protection and that the RBD as well as StabS proteins used in our study can be applied for sorting B cells and generating neutralizing monoclonal antibodies. Surveillance of anti-RBD and anti-StabS antibody responses therefore represent, as far as we analyzed, a reliable, simple, quick and high-throughput compatible alternative for any type of state of the art neutralization assay such as standard plaque reduction or lentiviral- and VSV-derived pseudotype assays. Such robust correlates will gain further importance once we start determining thresholds for protective immunity as a result of natural infection and, more importantly, following vaccination.

## Electronic supplementary material

Below is the link to the electronic supplementary material.

Supplementary file1 (DOCX 2395 kb)
