# Supplementary material for: A highly specific and sensitive serological assay detects SARS-CoV-2 antibody levels in COVID-19 patients that correlate with neutralization
Source: Infection. 2020 Aug 21;49(1):75–82. doi: 10.1007/s15010-020-01503-7 (PMC7441844; doi:10.1007/s15010-020-01503-7)
Supplement: Supplementary file 1 — Supplementary file1 (DOCX 2395 kb) [file 15010_2020_1503_MOESM1_ESM.docx]

**Supplementary figures, tables and methods**

**A highly specific and sensitive serological assay detects SARS-CoV-2 antibody levels in COVID-19 patients that correlate with neutralization**

David Peterhoff^1^*, Vivian Glück^2^, Matthias Vogel^2^, Philipp Schuster^1^, Anja Schuetz^1^, Philip Neubert^2^, Veruschka Albert^2^, Stefanie Frisch^2^, Mara Kiessling^2^, Philip Pervan^2^, Maren Werner^1^, Nicole Ritter^2^, Leon Babl^2^, Maria Deichner^2^, Frank Hanses^3,4^, Matthias Lubnow^5^, Thomas Müller^5^, Dirk Lunz^6^, Florian Hitzenbichler^3^, Franz Audebert^7^, Viola Hähnel^8^, Robert Offner^8^, Martina Müller^9^, Stephan Schmid^9^, Ralph Burkhardt^10^, Thomas Glück^11^, Michael Koller^12^, Hans Helmut Niller^1^, Bernhard Graf^6^, Bernd Salzberger^3^, Jürgen J. Wenzel^2^, Jonathan Jantsch^1,2^, André Gessner^1,2^, Barbara Schmidt^1,2^*^+^, and Ralf Wagner^1,2^*^+^

*corresponding authors; ^+^contributed equally

Lead corresponding author: [david.peterhoff@ur.de](mailto:david.peterhoff@ur.de)

^1^ Institute for Medical Microbiology and Hygiene, University of Regensburg, Regensburg, Germany

^2^ Institute for Clinical Microbiology and Hygiene, University Hospital Regensburg, Regensburg, Germany

^3^ Department for Infection Control and Infectious Diseases, University Hospital Regensburg, Regensburg, Germany

^4^ Emergency Department, University Hospital Regensburg, Regensburg, Germany

^5^ Department of Internal Medicine II, University Hospital Regensburg, Regensburg, Germany

^6^ Department of Anesthesiology, University Hospital Regensburg, Regensburg, Germany

^7^ Praxiszentrum Alte Mälzerei, Regensburg, Germany

^8^ Institute of Clinical Chemistry and Laboratory Medicine, Transfusion Medicine, University Hospital Regensburg, Regensburg

^9^ Department of Internal Medicine I, University Hospital Regensburg, Regensburg, Germany

^10^ Institute of Clinical Chemistry and Laboratory Medicine, University Hospital Regensburg, Regensburg, Germany

^11^ Kreisklinik Trostberg, Trostberg, Germany

^12^ Center for Clinical Studies, University Hospital Regensburg, Regensburg, Germany

**
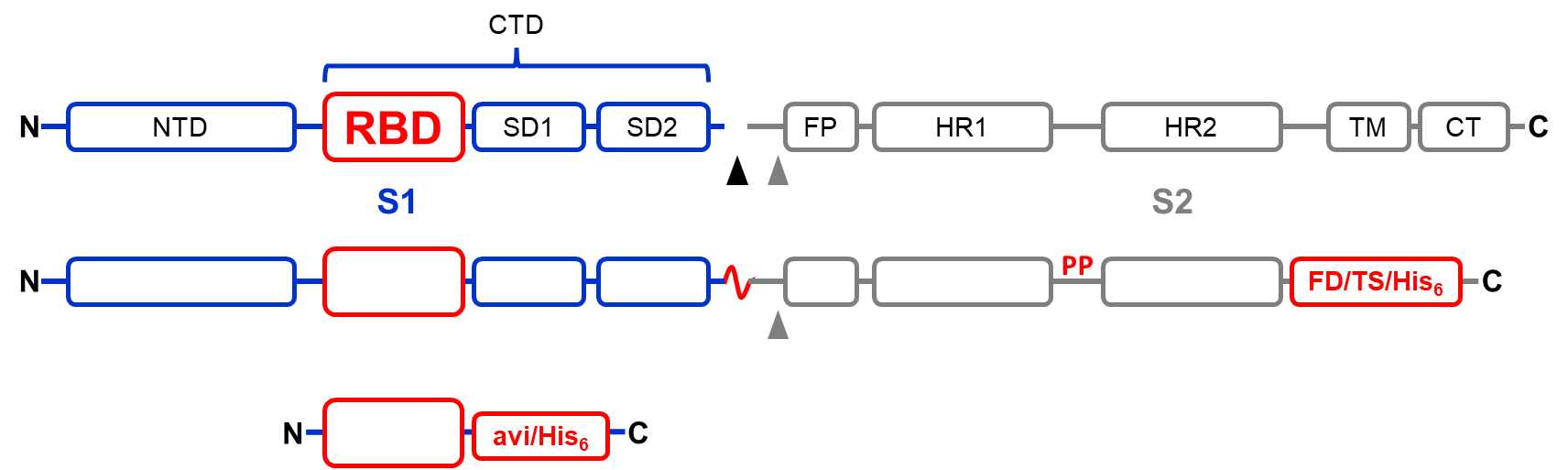
**

**Fig. S1: Topology diagram of SARS-CoV-2 S protein and its synthesized variants.** NTD, N-terminal domain of S1; CTD, C-terminal domain of S1; SD1/2, subdomain 1 and 2 of CTD; FP, fusion peptide; HR1/2, heptad repeats 1 and 2; TM, transmembrane domain; CT, cytoplasmic tail; FD, foldon tag; TS, twinstrep tag; His_6_, hexahistidine tag; avi, avi tag; PP, stabilizing two proline substitution. Upper diagram: wildtype; middle diagram: StabS; lower diagram: receptor-binding domain (RBD). Triangles symbolize primary and secondary cleavage sites.

**
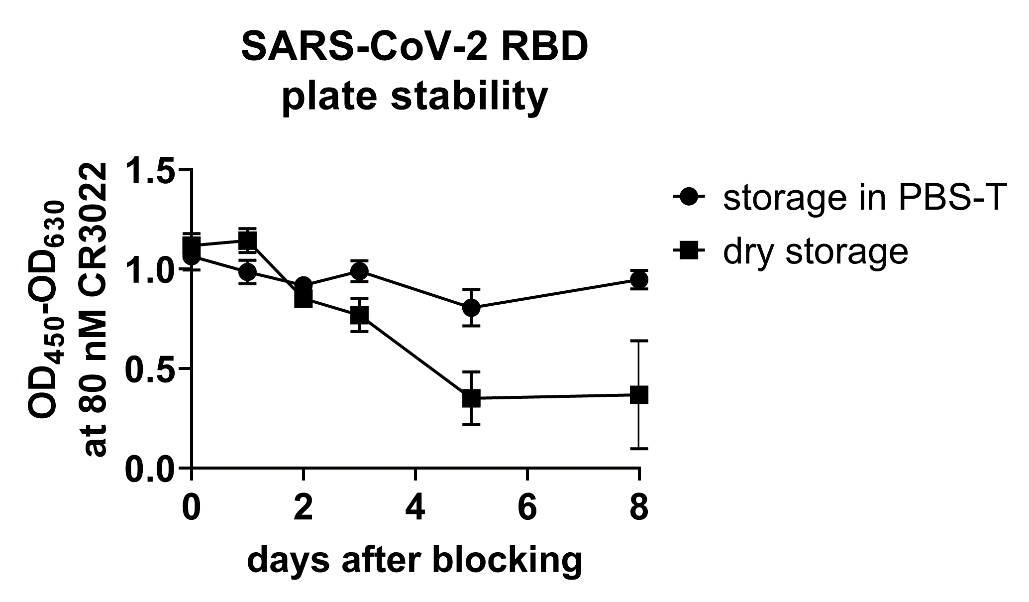
**

**Fig. S2: SARS-CoV-2 RBD ELISA plate stability.** Plates were coated over night and blocked. Wells were either stored dry or in 200 µl PBS-T at room temperature and binding of the structure-dependent CR3022 antibody was measured at saturating concentrations at different time points (N=8, mean and standard deviation).


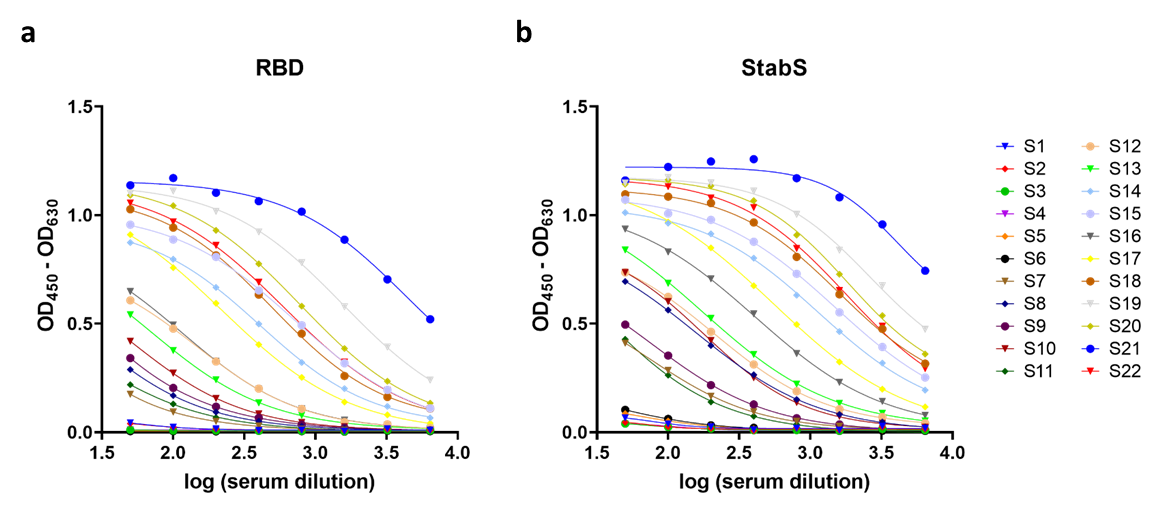


**Fig. S3: ELISA titrations of 22 COVID-19 patient sera displaying differential serum reactivity against the RBD or StabS.** Sera were measured in two-fold serial dilutions starting at 1:50 serum dilution. Reactivity against (a) RBD and (b) StabS is depicted.


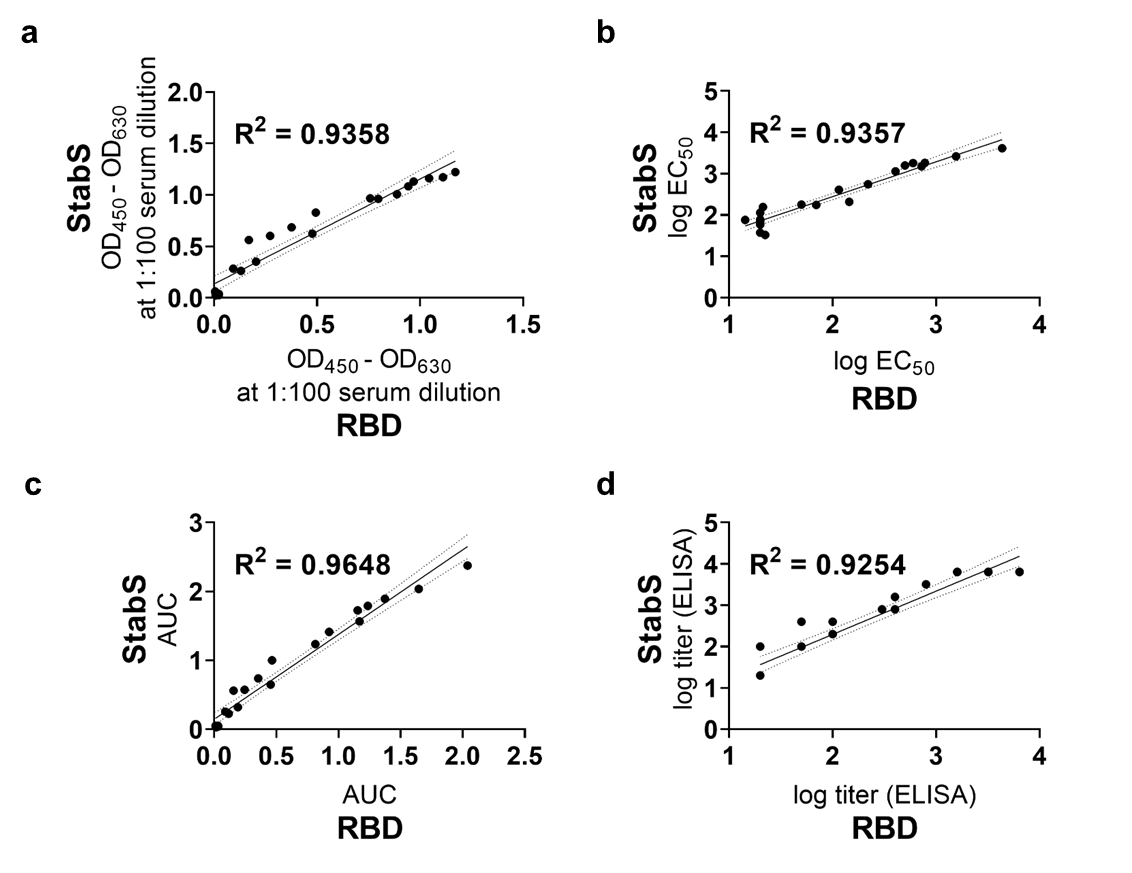


**Fig. S4: Validation of the ELISA.** Correlation of parameters from ELISA measurements of 22 representative sera displaying different SARS-CoV-2 IgG-reactivities. (a) Optical densities at 1:100 serum dilution, (b) log EC_50_ values, (c) area under the curve, and (d) log titers. Coefficients of determination (R^2^), linear regression line (solid line) and 95 % confidence intervals (dashed line) are shown in the diagrams.


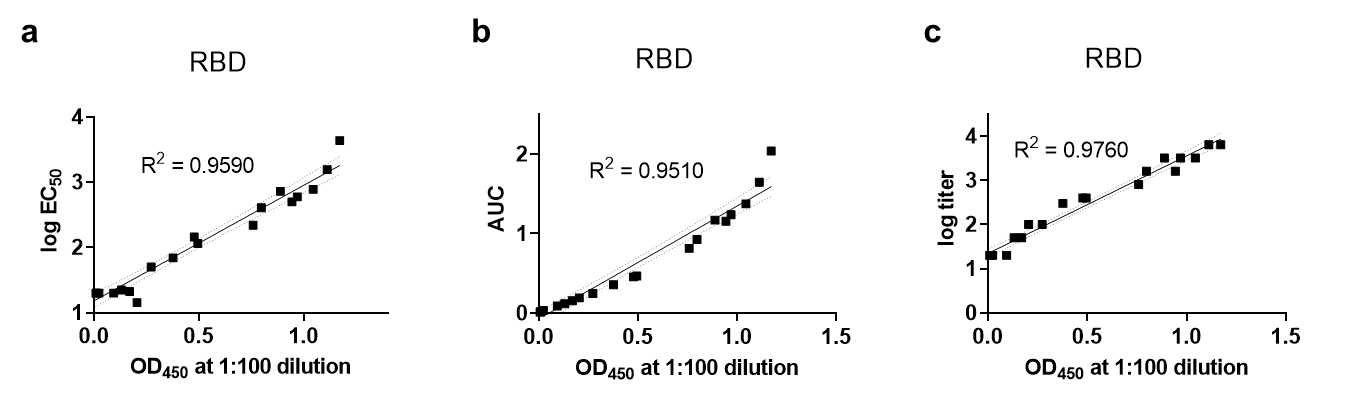


**Fig. S5: Validation of the ELISA.** Correlation of the OD values from the ELISA measured against RBD with the corresponding log EC_50_ (a), area under the curve (b) and log titer values (c) for the 22 reference sera. Coefficients of determination (R^2^), linear regression line (solid line) and 95 % confidence intervals (dashed line) are shown in the diagrams.


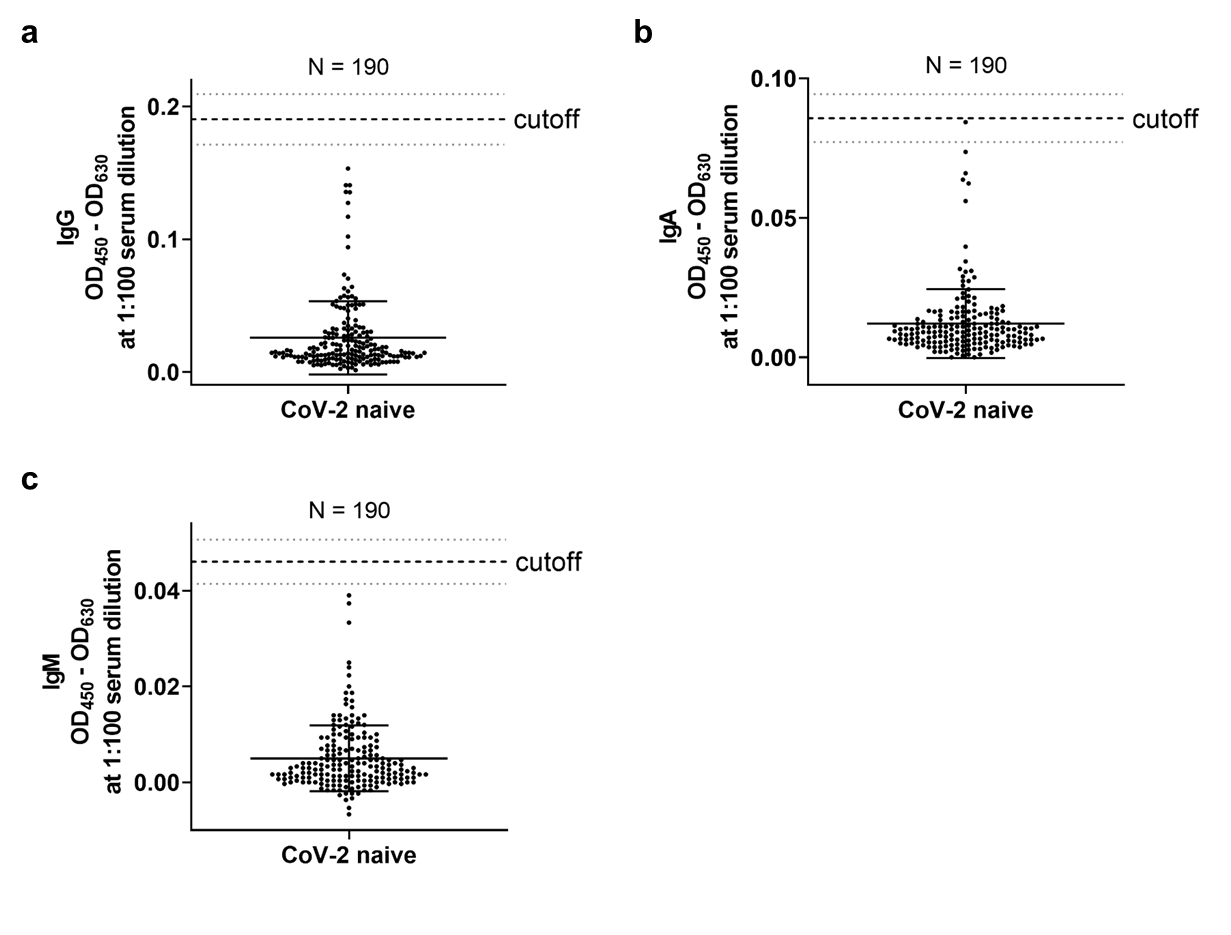


**Fig. S6: Determination of the cutoff value.** For determination of a cutoff value for (a) IgG, (b) IgA, and (c) IgM 190 sera obtained in July and August 2017 as well as July and August 2019 (SARS-CoV-2 naive) were measured at 1:100 dilution. Mean and standard deviation is shown. Cutoff is set at the mean plus six standard deviations. Samples were labeled „borderline“ within ± 10% of the cutoff value.


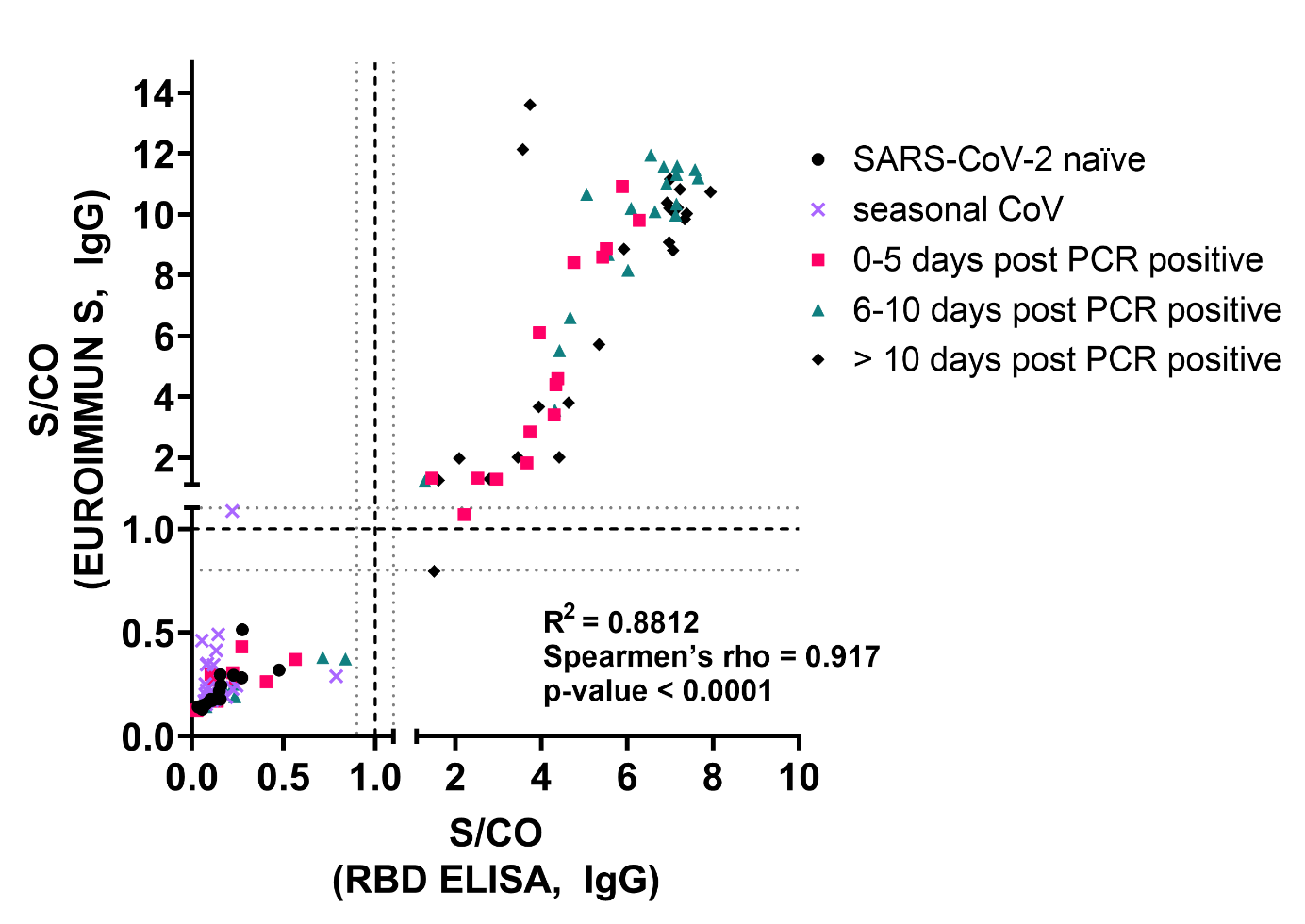


**Fig. S7: IgG ELISA data from RBD ELISA and EUROIMMUN S ELISA.** Correlation of S/CO value obtained from measuring a panel of 15 SARS-CoV-2 naïve patient sera (circle), 30 seasonal CoV positive sera (cross), 25 sera from 0-5 days post positive SARS-CoV-2 RT-qPCR (square), 25 sera from 6-10 days post positive SARS-CoV-2 RT-qPCR (triangle) and 25 sera from > 10 days post positive SARS-CoV-2 RT-qPCR (rhombic). Coefficients of determination (R^2^) Spearmen’s rho and p-value for all values is shown in the graph.


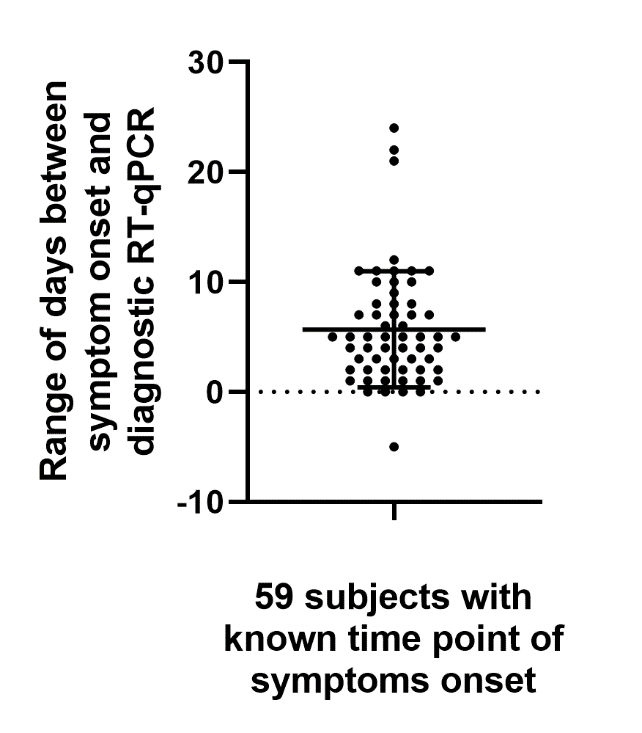


**Fig. S8: Period between the time point of symptoms onset and diagnostic RT-qPCR.** For 59 subject of the cohort, time point of symptoms onset and RT-qPCR data was available. Mean and standard deviation are shown.


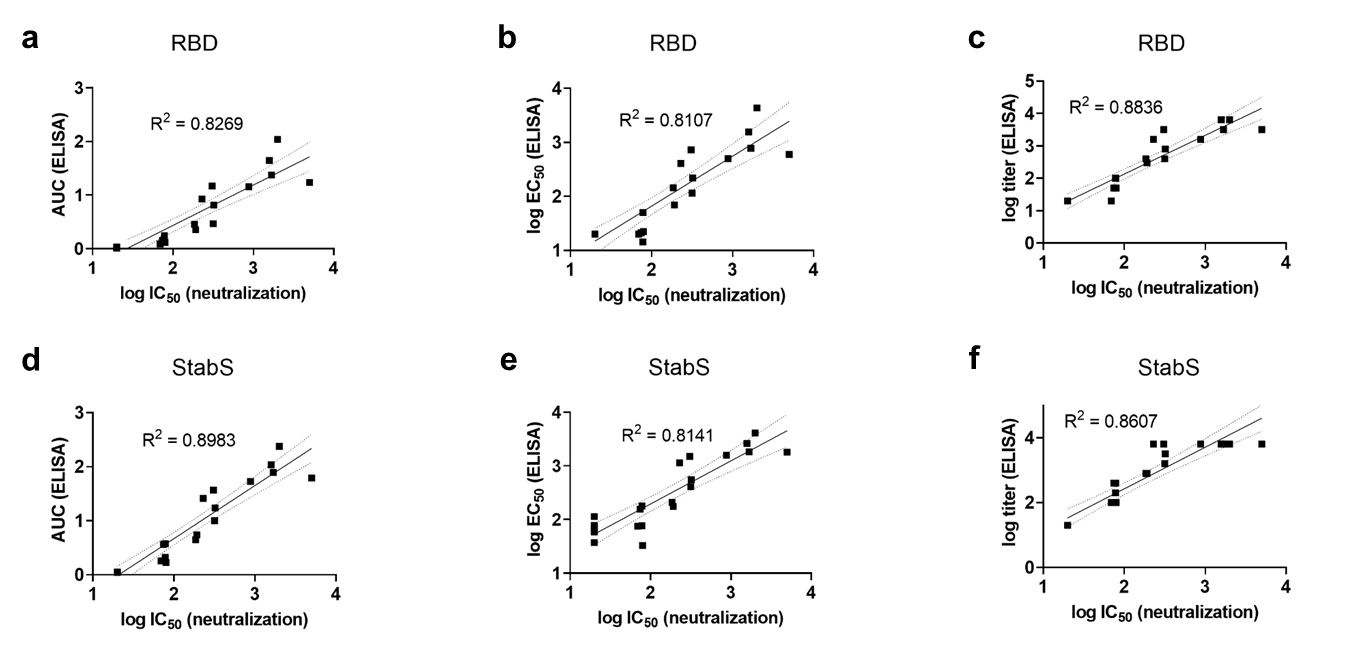


**Fig. S9: Correlation of ELISA and virus neutralization data.** Correlation of different ELISA parameters with log IC_50_ values obtained from the neutralization experiments for a panel of 22 reference sera (a-c) using RBD as antigen or (d-f) using StabS as antigen. Coefficients of determination (R^2^), linear regression line (solid line) and 95% confidence intervals (dashed line) are shown in the diagrams.


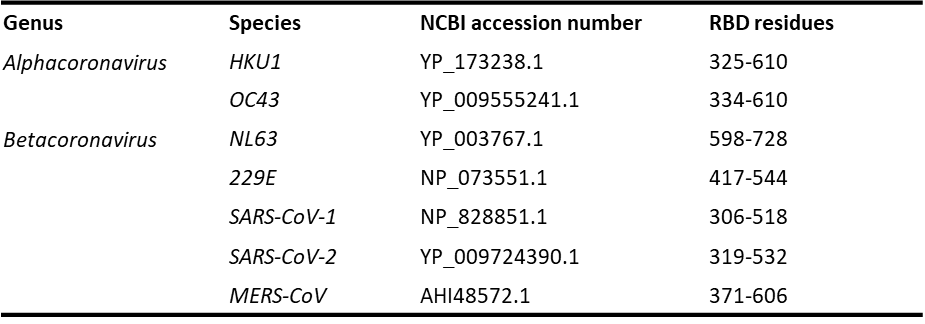


**Table S1: S-Protein sequences used for sequence analysis and gene synthesis.**

**Table S2: Results from line blot analysis of 43 sera from patients with PCR proven seasonal CoV infection.** FTD Respiratory pathogens 21: PCR result. Visual inspection: +++ strong signal; ++ intermediate signal; + weak signal; +/- signal below cutoff; - no signal. *recom*Scan: signal per cutoff from automated line blot evaluation software. One naive control serum from summer 2019 (naive) and two SARS-CoV-2 positive sera (weak and strong positive) were included in the analysis.

**Supplementary Materials and Methods:**

Design of recombinant proteins

S-protein sequences used for sequence analysis and gene synthesis are deposited in Table S1. Phylogenetic trees were generated by Jalview [1] and visualized by Dendroscope [2] using multiple sequence alignments calculated with Clustal Omega and Muscle [3].

Human codon usage-optimized sequences were synthesized by GeneArt AG (part of Thermo Fisher Scientific Inc.). The SARS-CoV-2 RBD was cloned into a modified pcDNA5/FRT/TO encoding a minimal N-terminal tPA signal peptide (sequence: MDAMKRGLCCVLLLCGAVFVSPSAA) and a C-terminal avi-hexahistidine tag (sequence: GGSGLNDIFEAQKIEWHEGSHHHHHH). The SARS-CoV-2 StabS protein was cloned into a pcDNA3.1(+) vector essentially as described by Wrapp *et al.* [4]. Briefly, the SARS-CoV-2 coding sequence was fused to the minimal N-terminal tPA signal peptide for efficient secretion of the protein and modified by a S1/2 cleavage site knock out (RRAR at residue 682-685 replaced by GSAS), a proline-proline substitution (KV at residue 986-987 replaced by PP), a truncation at position 1208 followed by a foldon-tag, a human rhinovirus type 3C protease site, a His_8_- and a twin-strep-tag (Fig. S1).

Heavy chain and light chain variable region sequences of antibody CR3022 were retrieved from NCBI accession numbers DQ168569 and DQ168570. The sequences were synthesized and fused to the human IgG1 heavy chain Fc region (GenBank accession P01857) and the human IgG light chain kappa constant region sequence (GenBank accession AAD29610.1), respectively. Both antibody sequences were cloned into a pcDNA5/FRT/TO derivate providing a murine IgG1 signal peptide sequence for efficient translocation into the secretory pathway.

Protein structure analysis and visualization

Protein structures were visualized and analyzed by Pymol (LLC Schrodinger) using the structural data from the Protein Data Bank repository, particularly involving entry codes 6vsb and 6m17.

Protein production and purification

Plasmids encoding the SARS-CoV-2 RBD and SARS-CoV-2 StabS were transfected into Expi293 cells (Thermo Fisher Scientific) in different scales using the commercial ExpiFectamine^TM^ system as a transfection and expression booster reagent system with 1 µg DNA per ml cell culture according to the manufacturer’s recommendations. Plasmids coding for light chain and heavy chain of CR3022 were co-transfected with equal amounts (w/w) of DNA using the same method and conditions. Protein expression was carried out for 5 days. Supernatants were harvested by centrifuging twice for 20 min at 1000 rcf and 8 °C, filtrated (0.2 µm) and preserved by adding 0.05% (w/v) sodium azide.

Spike protein variant supernatants were loaded twice onto a 5 ml immobilized metal chelate affinity chromatography column (HisTrap Excel , GE Healthcare) with a flow rate of 1 ml/min and subsequently recirculated over night at 4 °C. After washing with 100 ml Dulbecco’s Phosphate Buffered Saline (PBS, Sigma) containing 10 mM imidazole (Sigma) at a flow rate of 1 ml/min, protein was eluted over a linear gradient of 10-500 mM imidazole in PBS using a FPLC device (ÄKTA, GE Healthcare). Peak fractions were collected and analyzed for the presence of the target protein by sodium dodecyl sulfate polyacrylamide gel electrophoresis (SDS-PAGE) under reducing conditions. Fractions containing the target protein were pooled and buffer exchanged to PBS by Sephadex G-25 desalting columns (GE Healthcare). Protein solutions were concentrated to approximately 1-2 mg/ml by ultrafiltration devices (Amicon Centrifugal Filters, Merck Millipore) with a molecular weight cutoff of 10 kDa for RBD and 30 kDa for StabS.

CR3022 supernatants were loaded onto a protein A column (1 ml HiTrap MabSelect SuRe, GE Healthcare) as described for the spike proteins. The column was washed with 50 ml PBS and the antibody was eluted by a pH shift in 100 mM glycine pH 3.2. The eluate was immediately neutralized by addition of 1 M Tris/HCl pH 7.0 and buffer exchanged to PBS by Sephadex G-25 desalting columns (GE Healthcare).

Polyacrylamide gel electrophoresis

For SDS-PAGE analysis 10 µg protein was loaded onto a precast acrylamide gradient gel (SERVAGel TG PRiME, 8-16% polyacrylamide, Serva), run for 1 h at 200 Volts, and stained with Coomassie Brilliant Blue G-250 Dye (ThermoFisher)‎.

Analytical size exclusion chromatography

For analytical size exclusion gel electrophoresis, 25 µg protein was loaded to a Superdex 200 Increase 10/300 GL column (GE Healthcare) operated on an HPLC system (Agilent 1100, Agilent) in PBS at a flow rate of 0.75 ml/min.

Enzyme-linked Immunosorbent Assay

Nunc MaxiSorp plates (Thermo Fisher Scientific) were coated with 50 µl of a 2 µg/ml dilution of RBD or StabS in PBS (Sigma) at 4 °C over night. Plates were blocked with 200 µl 5% fat free milk powder (Heirler) in PBS with 0.1% Tween 20 (Caelo) for 1 h at room temperature (RT). After washing three times (HydroFlex Microplate Washer, Tecan) with 200 µl PBS containing 0.1% Tween 20 (PBS-T), 50 µl of single (1:100) or serial dilutions (1:50, 1:100, 1:200, 1:400, 1:800, 1:1,600, 1:3,200, 1:6,400) of serum in 1% fat free milk powder in PBS-T were added and the plates were incubated for 1 h at RT. Plates were washed ten times with 200 µl PBS-T and secondary antibody horseradish peroxidase conjugate was added in 50 µl PBS-T. We used polyclonal rabbit anti-human IgG/HRP conjugate (Agilent, Dako) at 1:5,000 dilution, polyclonal rabbit anti-human IgA/HRP conjugate (Agilent, Dako) at 1:4,000 dilution and polyclonal goat anti-human IgM/HRP conjugate (Sigma) at 1:3,000 dilution. For IgA and IgM measurements, IgG was absorbed before adding the serum dilution to the wells by pre-incubation with RF-Absorbent (Virion\Serion GmbH) to avoid false positive or false negative results due to autoreactive IgM species or antibody competition. After 1 h incubation at RT, plates were washed ten times with 200 µl PBS-T. Substrate solution A (30 mM tri-potassium citrate monohydrate [Sigma-Aldrich] pH 4.1) and substrate solution B (10 mM 3,3',5,5'-tetramethylbenzidine [Roth], 80 nM hydrogen peroxide in 10% v/v acetone and 90% v/v ethanol) were freshly mixed at a 20:1 (v/v) ratio. 50 µl substrate solution was added to each well, incubated for 4 min at RT, and stopped by adding 25 µl of 1.0 N sulfuric acid. Optical density was determined after 5 minutes in a plate reader by measuring at 450 nm (OD_450_) and 630 nm (OD_630_) (Microplate Reader Model 680, Bio-Rad) in three technical replicates. For evaluation, OD_630_ values were subtracted from OD_450_ values as background.

Virus isolation

SARS-CoV-2 was isolated from respiratory specimen using the lung carcinoma-derived cell line A549, the hepatocarcinoma-derived cell line Huh-7, and African green monkey-derived kidney epithelial cells (Vero). Cells were propagated in Dulbecco’s Modified Eagle‘s Medium supplemented with 10% heat-inactivated fetal calf serum (Sigma-Aldrich), 90 U/ml streptomycin, 0.3 mg/ml glutamine, 200 U/ml penicillin. To prevent fungal infection, 2.5 µg/ml amphotericin B (PAN Biotech) was added when respiratory specimen were inoculated onto non-confluent cell layers. At peak of virus replication, viral supernatants were harvested, filtered through 0.22 µm pore sizes, and stored frozen in aliquots at -80 °C.

Viral loads were determined using a quantitative SARS-CoV-2 real-time RT-PCR (RT-qPCR, [5]). The 50% tissue culture infective dose (TCID_50_) was determined using the method of Reed and Munch [6].

Virus neutralization assay

Vero cells were plated in 96-well flat bottom plates at 15,000 cells/well and infected using a multiplicity of infection (MOI) of 0.05. Prior to infection, the virus inoculum was incubated with serial dilutions of serum samples (1:20, 1:80, 1:320, 1:1,280, 1:5,120, 1:20,480) for one hour and then added to the cells. To remove the input virus, media were exchanged 12-24 hours post plating and dilutions of serum samples were replenished. Two days post infection, viral loads were determined in cell culture supernatants using RT-qPCR, and half maximal inhibitory concentrations (IC_50_) were calculated using GraphPad Prism version 8.4.2.

**Supplemantary** **References**

1. Waterhouse AM, Procter JB, Martin DM, Clamp M, Barton GJ. Jalview Version 2--a multiple sequence alignment editor and analysis workbench. Bioinformatics. 2009;25(9):1189-91. doi:10.1093/bioinformatics/btp033.

2. Huson DH, Scornavacca C. Dendroscope 3: an interactive tool for rooted phylogenetic trees and networks. Syst Biol. 2012;61(6):1061-7. doi:10.1093/sysbio/sys062.

3. Madeira F, Park YM, Lee J, Buso N, Gur T, Madhusoodanan N et al. The EMBL-EBI search and sequence analysis tools APIs in 2019. Nucleic Acids Res. 2019;47(W1):W636-W41. doi:10.1093/nar/gkz268.

4. Wrapp D, Wang N, Corbett KS, Goldsmith JA, Hsieh CL, Abiona O et al. Cryo-EM structure of the 2019-nCoV spike in the prefusion conformation. Science. 2020;367(6483):1260-3. doi:10.1126/science.abb2507.

5. Corman VM, Landt O, Kaiser M, Molenkamp R, Meijer A, Chu DK et al. Detection of 2019 novel coronavirus (2019-nCoV) by real-time RT-PCR. Eurosurveillance. 2020;25(3):2000045. doi:doi:<https://doi.org/10.2807/1560-7917.ES.2020.25.3.2000045>.

6. Reed LJ, Muench, H. A simple method of estimating fifty percent endpoints. The American Journal of Epidemiology. 1938;27(3):493-7.
